# Application of dissociation curve analysis to radiation hybrid panel marker scoring: generation of a map of river buffalo (*B. bubalis*) chromosome 20

**DOI:** 10.1186/1471-2164-9-544

**Published:** 2008-11-17

**Authors:** Kelli J Kochan, M Elisabete J Amaral, Richa Agarwala, Alejandro A Schäffer, Penny K Riggs

**Affiliations:** 1 Department of Animal Science, Texas A&M University, College Station, Texas, USA; 2 Departamento de Biologia, Instituto de Biociências, Letras e Ciências Exatas (IBILCE), Universidade Estadual Paulista (UNESP), Campus de São José do Rio Preto, São Paulo, Brasil; 3 National Center for Biotechnology Information, National Institutes of Health, Department of Health and Human Services, Bethesda, Maryland, USA

## Abstract

**Background:**

Fluorescence of dyes bound to double-stranded PCR products has been utilized extensively in various real-time quantitative PCR applications, including post-amplification dissociation curve analysis, or differentiation of amplicon length or sequence composition. Despite the current era of whole-genome sequencing, mapping tools such as radiation hybrid DNA panels remain useful aids for sequence assembly, focused resequencing efforts, and for building physical maps of species that have not yet been sequenced. For placement of specific, individual genes or markers on a map, low-throughput methods remain commonplace. Typically, PCR amplification of DNA from each panel cell line is followed by gel electrophoresis and scoring of each clone for the presence or absence of PCR product. To improve sensitivity and efficiency of radiation hybrid panel analysis in comparison to gel-based methods, we adapted fluorescence-based real-time PCR and dissociation curve analysis for use as a novel scoring method.

**Results:**

As proof of principle for this dissociation curve method, we generated new maps of river buffalo (*Bubalus bubalis*) chromosome 20 by both dissociation curve analysis and conventional marker scoring. We also obtained sequence data to augment dissociation curve results. Few genes have been previously mapped to buffalo chromosome 20, and sequence detail is limited, so 65 markers were screened from the orthologous chromosome of domestic cattle. Thirty bovine markers (46%) were suitable as cross-species markers for dissociation curve analysis in the buffalo radiation hybrid panel under a standard protocol, compared to 25 markers suitable for conventional typing. Computational analysis placed 27 markers on a chromosome map generated by the new method, while the gel-based approach produced only 20 mapped markers. Among 19 markers common to both maps, the marker order on the map was maintained perfectly.

**Conclusion:**

Dissociation curve analysis is reliable and efficient for radiation hybrid panel scoring, and is more sensitive and robust than conventional gel-based typing methods. Several markers could be scored only by the new method, and ambiguous scores were reduced. PCR-based dissociation curve analysis decreases both time and resources needed for construction of radiation hybrid panel marker maps and represents a significant improvement over gel-based methods in any species.

## Background

In addition to quantification of mRNA after reverse transcription, real-time quantitative polymerase chain reaction (qPCR) technology has been utilized for diverse applications including detection and quantification of bacterial or viral pathogens [1,2], detection of mRNA splice variants [3], and quantification of transgene copy numbers [4]. Fluorescence-based dissociation curve analysis has been used for detection of DNA sequence polymorphisms from large deletions [5] to single nucleotide polymorphisms [6].

Since development of methods to construct marker maps via radiation hybrid (RH) somatic cell culture panels [7-9], RH mapping has become a method of choice. For large mammals, the generation interval and animal husbandry costs make construction of genetic maps expensive and lengthy endeavors. Many livestock species, particularly those important in countries where resources for genetic evaluation and improvement are scarce, can benefit greatly from continued mapping efforts. For species whose genomes have been sequenced, RH mapping remains a useful tool to aid assembly of sequence scaffolds [10]. Methods for construction of whole-genome RH mapping panels have been described in detail previously [11], and to date, whole-genome RH mapping panels have been produced for livestock animals including pig [12] chicken [13], horse [14], cattle [15,16], sheep [17] and river buffalo [18]. As livestock, river buffalo (*Bubalus bubalis*) provide a source of meat, dairy products, and draught power throughout the world including Asia, South America, the Mediterranean, and other regions (reviewed in Boyazaglu [19], Bernardes, [20] and Cruz [21]). This species can benefit from the same selection and mapping efforts that have been applied to domestic cattle [22].

Once an RH panel is constructed, development of maps involves three major steps: 1) marker development, 2) marker scoring, and 3) computation of optimal marker order. The availability of genome sequences from related organisms has speeded marker development [23], and new software for computing RH maps [24] makes computation fast, leaving marker scoring as the major time bottleneck in construction of RH maps. Gel-based methods (e.g., [25,26]) are labor intensive, and in cases where PCR products can be amplified from both donor and recipient DNA, products must usually be differentiated on the basis of size. Here we demonstrate the novel application of qPCR technology and dissociation curve analysis as a rapid and robust method for typing radiation hybrid panel DNA, and construct a map of river buffalo (*Bubalus bubalis*) chromosome 20 (BBU20) as proof of principle.

## Results

### Initial marker testing

River buffalo chromosome 20 is orthologous to domestic cattle (*Bos taurus*) chromosome 21 (BTA21; [27]). Significant sequence conservation between river buffalo and cattle allows new buffalo maps to be generated from bovine markers [28]. To rapidly generate a map of BBU20, we took advantage of the existing published BTA21 marker map to identify PCR primers that could be used for mapping in buffalo. We chose primers for 65 markers or genes that were derived from cattle sequence and had been previously mapped to BTA21. To determine suitability for RH panel typing, we screened primer pairs by PCR-amplification of DNA from river buffalo (the RH panel donor animal), A23 hamster (the RH panel recipient cell line), and cattle (positive control) fibroblast cells, followed by dissociation curve analysis of the products as described by Ririe et al. [29]. Of 65 marker primer sets, 31 markers were suitable for further analysis because they amplified river buffalo DNA well, exhibited dissociation curves that differed between the river buffalo and hamster products, and produced single product bands when checked by gel electrophoresis (Table 1). The remaining markers were discarded because they produced more than one river buffalo band, could not differentiate hamster and buffalo PCR products, or did not amplify. For more detailed explanation, see Additional File 1 and 2. Most of the primers used came from previously published bovine maps, and PCR optimization was not conducted because our objective was to implement a standardized qPCR protocol for screening and analysis to develop a rapid and efficient mapping method. We also compared data from manually prepared 96-well reactions to semi-automated robot-prepared 384-well reaction plates and obtained comparable results (not shown).

**Table 1:** Markers chosen for mapping analysis

Marker ID	Marker Type	Primer Sequence	Primer Reference
AGLA233	microsatellite	F: 5'-tgcaaacatccacgtagcataaata R: 5'-gcatgaacagccaatagtgtcatc	[40]
AKT-1	STS/gene	F: 5'-cacctgaccaagacgacagcat R: 5'-cgaggttccactcaaacgcatc	UniSTS: 277959; Accession# X61036
AKT-2	microsatellite	F: 5'-tgcccattcccagagccctgt R: 5'-cagctcgccccagggtgg	[41]
BM3413	microsatellite	F: 5'-tccctggtaaccaatgaattc R: 5'-caatggatttgaccctccc	[42]
BMC5221	microsatellite	F: 5'-agcaaggagaacaggcattc R: 5'-cttctttggcagcacagtttc	[42]
BMS1494	microsatellite	F: 5'-tctggagctgcaaaagacc R: 5'-aatggatgactcctggatgg	[43]
BMS1561	microsatellite	F: 5'-acccacatgttgggagg R: 5'-agggaaaggccaaagcac	Stone, 1996 (unpublished) Accession# G18764
BMS2382	microsatellite	F: 5'-agcacggagtcgttgtctg R: 5'-ccatctggacagaacgttacc	[44]
BMS868	microsatellite	F: 5'-tcatccaaccatctcatcct R: 5'-acatggaaacgaacctacattc	[43]
CC530547	STS/gene	F: 5'-tgatggattacctgatgcttcttgc R: 5'-tcaaccacagtcttgcttgctttc	UniSTS: 476716
CHGA	STS/gene	F: 5'-cccttgcctttcaacgattatct R: 5'-tcaggagtcctcagctttcacc	F: new design from Accession# NC_007319.2 (5199500352008294) R: UniSTS: 278198
DIK2116	microsatellite	F: 5'-cagccacaactggaactcg R: 5'-gggtcggttgcatcacat	[40]
DIK2367	microsatellite	F: 5'-tgctctatgaatcccaagctg R: 5'-cctcgttttatggctgtgct	[40]
DIK2586	microsatellite	F: 5'-ggacgctgacttggaaggta R: 5'-caccaacccttgtttccagt	[40]
DIK2821	microsatellite	F: 5'-cctttctgtcgtctcccttg R: 5'-tccttggagggttttgtcc	[40]
DIK2849	microsatellite	F: 5'-cacagacgagatcagctcca R: 5'-ccgataattgtccccaacag	[40]
DIK3001	microsatellite	F: 5'-ctcggggccaaaaaccaaaaccta R: 5'-tccgagataaagtacagaaagtcc	[40]
DIK3009	microsatellite	F: 5'-tggggcccggaggagtggtg R: 5'-gatgttcgagggctttct	[40]
DIK3023	microsatellite	F: 5'-tcttgccacctttggcttt R: 5'-gaggcgtgatgacgtgtcca	[40]
DIK4322	microsatellite	F: 5'-tccatagtgccagtgagctg R: 5'-ggagcgtccaaagataacca	[40]
DIK4894	microsatellite	F: 5'-ccagctttcttcctttacagtg R: 5'-caatccttggactgggaaga	[40]
GRP58	STS/gene	F: 5'-gaaactccattttgctgtag R: 5'-aacccacgctaacttgtaac	new design from Accession# NC_007319.2 (4901848149040683)
IDVGA-39	microsatellite	F: 5'-acggtgggaacatcttgtcacta R: 5'-ccagtattcttcctgcgaaaaatc	[45]
IGF1R	STS/gene	F: 5'-ggaacatggtggacgtggac R: 5'-gatgcgttggtgcgaatgta	new design from Accession# NC_007319.2 (93003629360647)
ILSTS054	microsatellite	F: 5'-gaggatcttgattttgatgtcc R: 5'-agggccactatggtacttcc	[46]
ILSTS092	microsatellite	F: 5'-gagaaactttgggctgctgc R: 5'-atggattgcttctgtggacc	[46]
MBIP	STS/gene	F: 5'-actattcactggctgaacttg R: 5'-atggaaggtgacgtgttg	UniSTS: 278723
MFGE8	STS/gene	F: 5'-ggcacaaccgtatcacc R: 5'-tccatcccagacctactcag	new design from Accession# NC_007319.2 (2021101820195989)
MULGE4	microsatellite	F: 5'-gcaacccttctgatgtcatgaacc R: 5'-aaaagcacaactcccctcaaatcc	[47]
RM151	microsatellite	F: 5'-cccagaggtgacaacatttccag R: 5'-gatccaccaaaaaccagctgga	[48]
SERPINA1	STS/gene	F: 5'-aagaacctgtatcactccgaagc R: 5'-tgtgtttgggtcaagaacctttac	new design from Accession# NC_007319.2 (5259062252599991)

### Comparison of dissociation curve and gel-based typing methods

**Figure 1:**
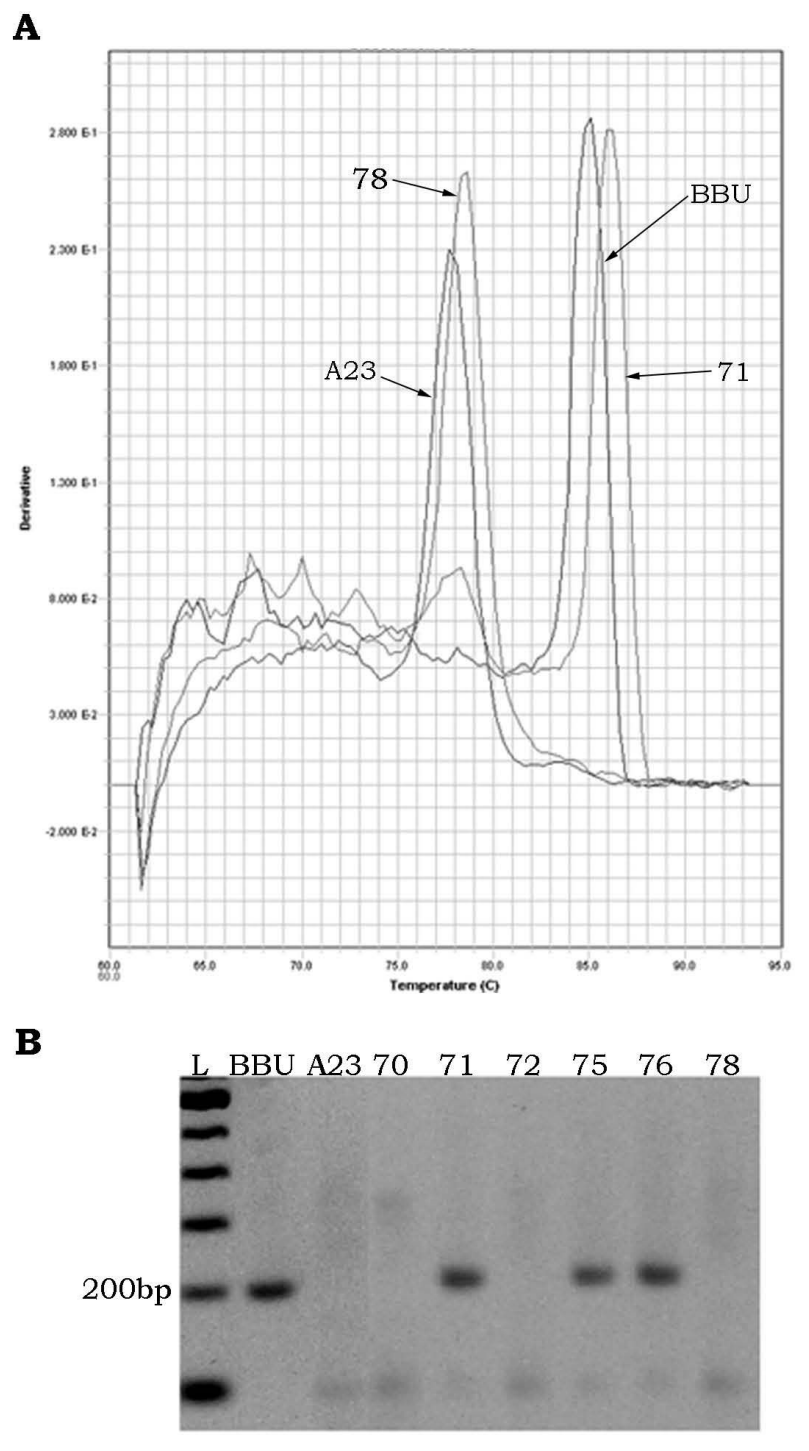
**Marker typing by qPCR and conventional method**. Dissociation curve (A) and gel image (B) of *DIK2849 *as an example of unambiguous +/- scoring of clones by both dissociation curve and agarose gel electrophoresis in buffalo (BBU), hamster (A23) and selected RH clones. In (A), the dissociation curve is plotted as the first derivative of fluorescence relative to temperature in the SDS software view. Clones #71 (positive for BBU DNA) and #78 (negative for BBU DNA) are indicated along with hamster negative control and buffalo positive control. Peaks are easily differentiated for scoring.

We typed 31 markers in a panel of 90 RH clones. Twenty-four markers were easily typed on the RH panel by both qPCR and gel electrophoresis, meaning that both agarose gels and dissociation curves resulted in clear differentiation of hamster and buffalo PCR products from individual hybrid clones. Of the 24 markers typed by both methods, 19 were scored unambiguously in all 90 RH clones and exhibited perfect accord between the two methods. Sample gel images and dissociation curves are shown in Figure 1. The remaining 5 markers were scored consistently by both methods in the majority of the RH clones, but 1 to 3 clones (out of 90) were scored as questionable (not clearly positive or negative for the product of interest) by one or the other method.

**Figure 2:**
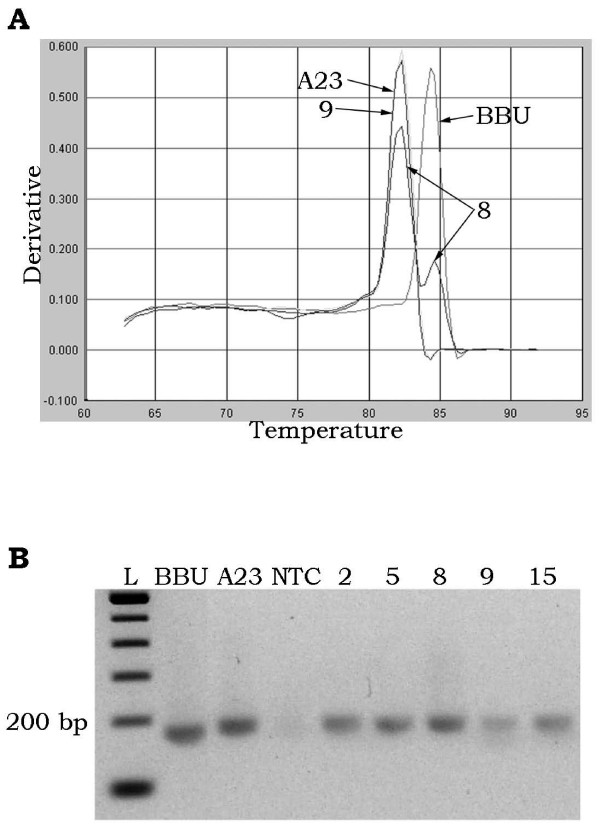
**Differentiation of similarly-sized products by qPCR**. Dissociation curve (A) and gel image (B) of *IGF1R *in buffalo (BBU), hamster (A23), no-template control (NTC) and selected RH clones. River buffalo and hamster products are indistinguishable on agarose gels, but are easily separated by differential dissociation curve analysis. Note that in clones positive for buffalo DNA (e.g. #8) hamster DNA is also amplified, as expected.

**Figure 3:**
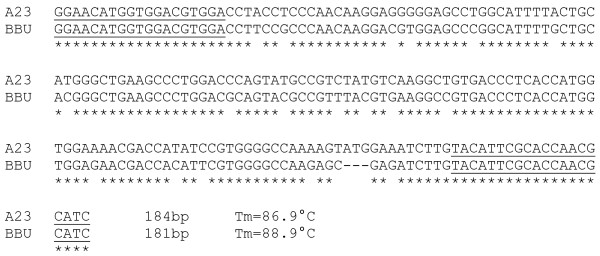
**Sequence variation of similarly-sized products**. CLUSTAL W (v. 1.83; [39]) alignment of *IGF1R *PCR product sequences of hamster (A23) and river buffalo (BBU). The primer sequences are underlined. Identical nucleotides are marked with an asterisk beneath. Product melting temperatures (T_m_) were estimated with Oligo 6 software, and while not identical to the melting curve generated by the SDS software, the relative differences in melt temperature between the buffalo and hamster products were similar for calculated and experimental values. Both methods indicated an approximate 2°C difference in T_m _which was sufficient for discrimination by dissociation curve analysis.

**Figure 4:**
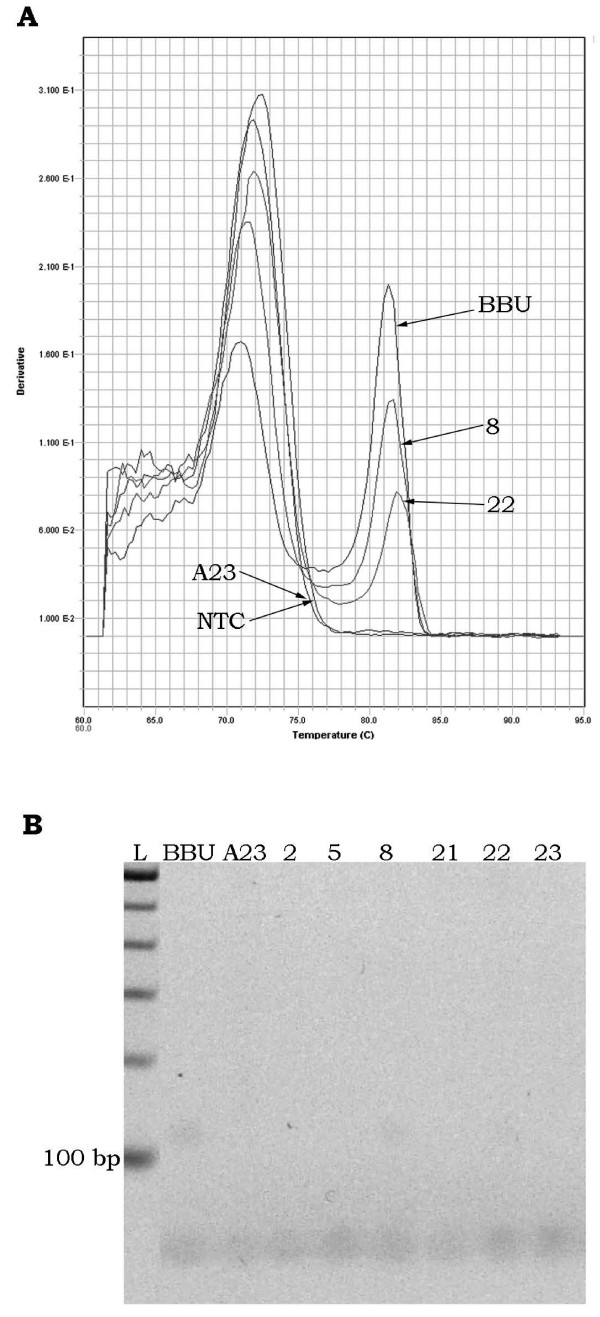
**Sensitivity of qPCR method**. Dissociation curve (A) and gel image (B) of *RM151 *in buffalo (BBU), hamster (A23) and selected RH clones. The left peak on the dissociation curve is presumed to be primer-dimer (~40 bp, as seen in the gel image), while the right peak is the target PCR product (barely visible or not at all in the gel image). This marker could not be scored by a gel-based method, but clones positive for buffalo DNA (#8, #22) are easily identified after dissociation curve analysis.

An additional 6 markers (25% increase) could be scored for mapping by the qPCR method alone under the described experimental reaction conditions. For 3 markers (*BMS1494*, *IGF1R *and *ILSTS092*), discrete and scorable dissociation curves were clearly seen, but agarose gel electrophoresis of PCR products resulted in bands that were indistinguishable between river buffalo and hamster (Figure 2). Sequence analysis of these markers [GenBank: BV727772, BV727773, BV727774, BV727775, BV727776 and BV727777] indicated that their PCR products actually differed in size by 1 to 6 bp between river buffalo and hamster. Sequence composition of the products also differed between the two species, resulting in different melting temperatures that could be readily scored (Figure 3). The remaining primer sets (*DIK2367*, *DIK4322 *and *RM151*) amplified so weakly that bands were too faint for reliable gel-based visual scoring, but signals observed by dissociation curve analysis were easily scored (Figure 4). In particular, *RM151 *produced a very strong primer-dimer band that overwhelmed the gel image, while the dissociation curve contained distinct peaks that readily distinguished the primer-dimer from the actual product.

**Figure 5:**
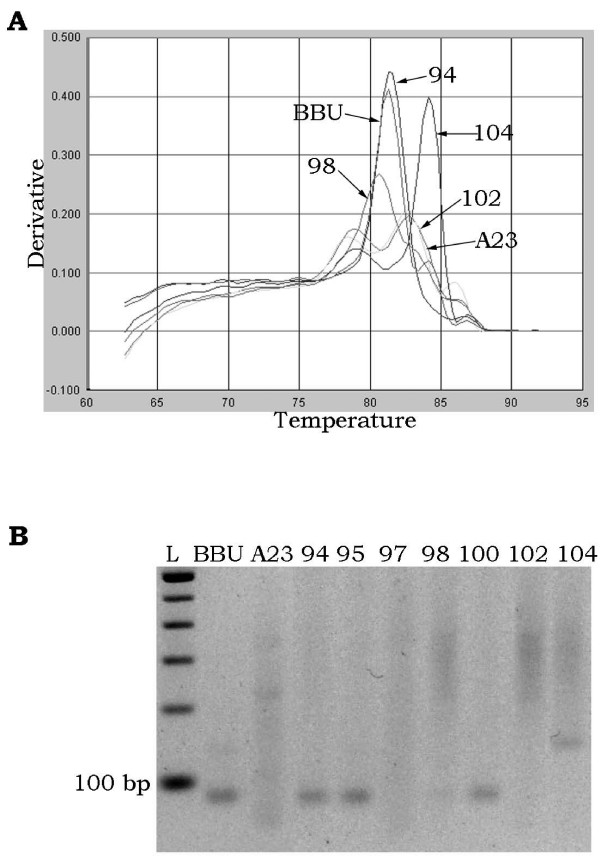
**Ambiguous qPCR typing of *AKT2***. Dissociation curve (A) and gel image (B) of *AKT2 *in buffalo (BBU), hamster (A23) and selected RH clones. Most "positive" clones (~90 bp target band present on gel) produced dissociation curves similar to the buffalo control (BBU, #94, #98) and most "negative" clones (target band absent on gel) produced dissociation curves similar to the hamster control (A23, #102); however, a number of clones produced intermediate or shifted dissociation curves that could not be scored convincingly (e.g. #104). Extraneous products and small target product size may have contributed to the variable dissociation curves for this marker.

The remaining marker, *AKT2*, was the only marker to produce scorable data by gel-based typing alone, as a number of the RH clones exhibited dissociation curves that did not match either of the controls. The *AKT2 *primer set produced a river buffalo band of about 90 bp (expected size based on the cattle product) in the clones that were scored positive by gel electrophoresis, while the negative clones exhibited one or more bands other than the target that presumably muddled the qPCR data (Figure 5).

### RH map construction

**Figure 6:**
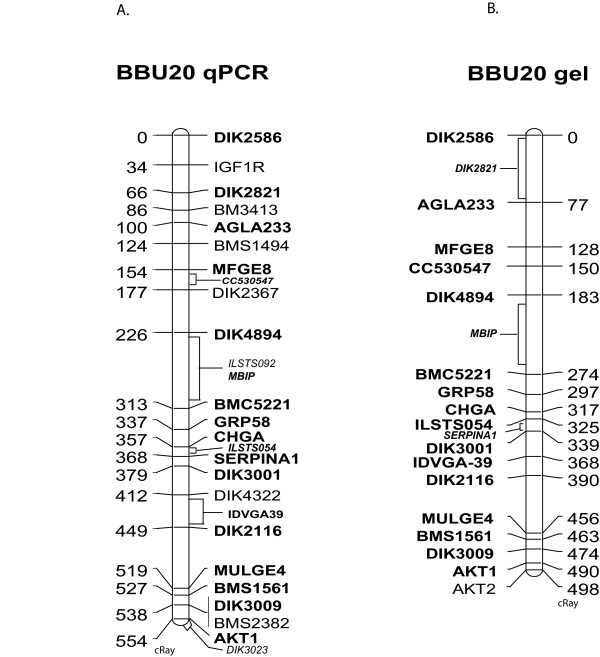
**BBU_5000 _RH map of BBU20 generated with dissociation curve data (A) and agarose gel electrophoresis data (B)**. Markers in plain text were placed only on one map, and those in bold were placed on both maps. Markers in italics were binned and not given a specific map location.

To further compare the two methods, we constructed separate RH maps of BBU20 from the qPCR- and gel-based scores. On the basis of qPCR scores, all 30 markers formed a single linkage group at LOD 7, of which 27 are included in the "qPCR map" (Figure 6A) that covers 92% of the MARC linkage map of cattle chromosome 21 . The three remaining markers (*RM151, BMS868, DIK2849*) could not be reliably placed in a specific interval of the framework map. For the "gel map," 20 of 25 markers were included, but it was necessary to reduce the LOD score cutoff to 6.5 to have those 20 markers form a single linkage group (Figure 6B). Among markers common to both maps, the marker order was identical; i.e., slight differences in the marker scores had no effect on the optimal map order.

## Discussion

Dissociation curve analysis is a viable alternative to conventional gel-based typing for RH mapping. In this case, primer sets chosen for RH mapping of river buffalo were based on sequence data from cattle, a related species. We expect that the method will be applicable to dissociation curve and high resolution melt analysis utilizing dyes such as SYTO 9, LCGreen, and EvaGreen in addition to the SYBR Green I utilized in this study. This mapping strategy allowed rapid genome mapping of our species of interest based on the existing high density marker map of the domestic cow. Markers for mapping were chosen more or less randomly to provide approximately even coverage of BTA21 microsatellite markers. We utilized a standardized PCR protocol without optimization to enable rapid, high-throughput gene mapping. Despite these constraints, we were able to type nearly half of the markers we tested, similar or slightly better than the success rate attained by our colleagues using conventional methods (e.g., [30]). Although greater success rates might have been achieved after PCR optimization for individual primer sets and a smaller proportion of microsatellite markers (e.g., [18]), our protocol reduced both time and resources spent identifying suitable markers by utilizing a single protocol.

Dissociation curve analysis is more robust than conventional typing. We typed 25% more markers by qPCR than by the gel-based method. While some of those markers might have yielded better gel-based data (i.e., stronger target bands) upon PCR optimization, qPCR data were produced without the use of additional time and resources necessary for optimization. Under conventional methods, some markers would have been discarded entirely without further consideration. The ability to differentiate PCR products not only by size, but also by sequence composition, is perhaps the greatest advantage of the qPCR method. Indeed, any means undertaken to increase the number of markers suitable for the gel-based map, such as a larger proportion of STS markers or coding genes, a requirement for well-designed primers (no hairpins or dimers, etc.), or optimization of the PCR protocol, would likely have led to a greater percentage of markers being included on the qPCR-based map.

The increased sensitivity of qPCR technology may reduce the incidence of scoring errors. Indirect evidence of more accurate scores is that 90% of the markers analyzed for the qPCR map ultimately comprised that map, compared to only 80% inclusion of analyzed markers in the linkage group for the gel map, even though the qPCR map used a higher LOD score threshold. Furthermore, among 25 markers typed on the agarose gels, individual clones were scored as questionable on a single PCR replicate due to faint bands or spurious bands of similar size to the target amplicon in 40 instances (~1.8% of all scores). In contrast, among 30 markers typed by qPCR, only 21 instances (~0.8% of all scores) of questionable results occurred in single replicates, generally due to shorter peaks for the buffalo product when the hamster product also amplified well. Conventional methods require duplicate typing to confirm accuracy and resolve questionable typing results. While qPCR technology does not completely eliminate the need for duplicate reactions, our observations indicate that qPCR typing may yield more accurate results than gel-based typing. Moreover, elimination of agarose gels removes the need for post-amplification handling and reduces opportunities for technical errors.

Typing data are generally scored as 1 (product present) or 0 (product absent), with questionable results scored as 2. Reducing the number of ambiguous 2 scores is important because addressing ambiguous scores computationally remains controversial. During RH map computation, different software packages use different mathematical formulations of the maximum likelihood criterion when ambiguous scores are present in the data [31]. In this study, the presence of clones scored as 2 in these BBU20 data sets did not affect the map order or which markers could be retained, but the presence of 2s in data for other maps we have computed has forced numerous markers to be dropped due to the ambiguity.

**Figure 7:**
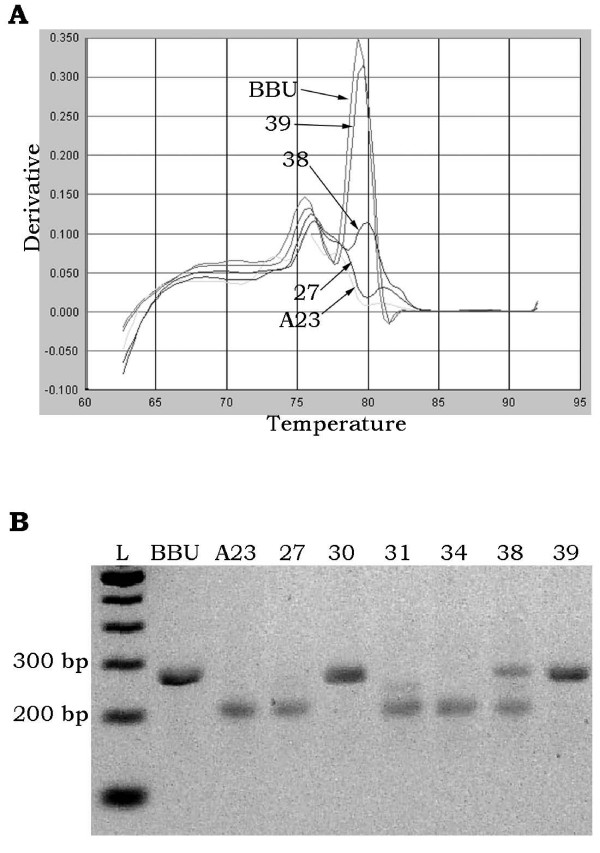
**Amplification of hamster product reduces buffalo product peak size**. Dissociation curve (A) and gel image (B) of *CC530547 *in buffalo (BBU), hamster (A23) and selected RH clones. Clone #38 produced a low, questionable peak on the dissociation curve, and unlike other positive clones (#30 and #39), exhibited the hamster band as well as the buffalo band in the gel.

Among more than 2000 individual scores (based on duplicate reactions) from the 24 markers scored by both qPCR and gel electrophoresis, only 10 scores were discordant between the two methods. Two and 3 individual clones were scored as questionable by qPCR for *DIK4894 *and *CC530547*, respectively, due to intermediate dissociation curves. On the gel, each of these markers had distinct river buffalo and hamster bands clearly distinguishable by size. Most RH clones exhibited one band or the other, and these were in perfect accord with the qPCR data, but the questionable clones exhibited both bands (Figure 7). Two clones were also scored as questionable by qPCR for *DIK2821*, along with one questionable curve for *IDVGA-39*; all were scored positive on the gels, but consistently exhibited weak bands in comparison to the other positive clones (data not shown). Finally, two clones were scored as questionable for *MFGE8 *by gel-based typing due to the consistent presence of a faint band at the expected size. Both clones were clearly negative by qPCR, so the gel bands were sequenced. Neither sequence matched the expected product; in fact, neither band produced clean sequence, indicating that dissociation curve analysis yielded more accurate results in this instance. In this experiment, with only 20 markers on the map, scoring those two clones as ambiguous did not affect the placement of *MFGE8 *on the map. As the river buffalo mapping project continues and the map becomes more dense, similar errors could change the statistically optimal marker order.

Dissociation curve analysis utilizes fewer resources than conventional typing. While the actual cost of typing a single marker by either method may vary greatly depending on choice of particular reagents and plastic ware, we applied similar criteria to reagent selection for both methods and found similar costs for PCR amplification (not shown, but details can be provided by the corresponding author upon request). The beauty of the qPCR method is that elimination of post-amplification procedures (i.e., gels) reduces the labor time required to type each marker. At the same time, expense and hazardous waste associated with agarose gels and ethidium bromide staining procedures is eliminated.

Other non-conventional PCR-based methods have been applied to RH mapping including single-strand conformational polymorphism (PCR-SSCP; [32]), where PCR products can be differentiated based on sequence composition, and amplified length polymorphism (AFLP; [33]), which has the advantage of producing markers without any prior sequence information for the species of interest, but results in essentially anonymous markers. However, these methods and related techniques require post-amplification separation and visualization of products by radioisotopic labeling [32] or silver staining [34], which necessitates additional handling and disposal of hazardous materials. Fluorescence labeling [35] reduces hazardous materials but still requires time-consuming procedures for separation and visualization of PCR products.

High-throughput methods will have great utility for map construction. For example, McKay et al [36] computed a dense bovine RH map with Illumina Golden Gate technology. Once the effort to make the Illumina array is completed, all the markers can be genotyped rapidly. Thus, the cost per marker decreases greatly, but once the array design is completed, one cannot easily add individual markers of interest. For techniques such as those used by McKay et al, or for application of "next generation" sequencing technology, or utilization of SNP microarrays, the cost per sample is relatively low. However, startup costs remain high, and the volume of data to be processed must also be considered. For many species still lacking maps, suitable array-based tool are simply unavailable, emphasizing the importance of alternative methods for genotyping and map building. In practice, we have found that application of some of these methods to RH mapping is costly and data interpretation is not completely straightforward at this time (Amaral et al, unpublished). In contrast, assuming available instrumentation, startup cost to run a single marker by qPCR is the same cost as equivalent PCR amplification, and likely more accessible to research groups involved in mapping species for which both gene maps and financial resources are currently limited.

The application of qPCR technology and fluorescence chemistry to DNA typing does impose limits on marker choice, particularly amplicon size. In this case, because SYBR Green I fluoresces upon laser excitation when bound to any double-stranded DNA, formation of primer-dimers may alter the dissociation curve of small (< 100 bp) PCR products. Anecdotal evidence and our experience indicate that SYBR-based dissociation curves appear to be most reliable for product sizes greater than 100 bp. Some of the odd dissociation curves we observed with *AKT2 *may have been influenced by the small PCR product size (~90 bp) of that marker. Indeed, it was the only marker that we attempted to type that was < 100 bp, and the only one that failed to produce clear, consistent results in the RH panel by qPCR. Large amplicons (> 500 bp) are not amplified as efficiently and may also produce inconsistent results. A brief survey of published RH maps indicates that PCR-based markers tend to fall in the range of 75–400 bp, with the majority ranging from 100–250 bp. In this experiment, the largest amplicon generated was ~290 bp, so we did not observe any deleterious effects due to large product size. Ultimately the success of qPCR and subsequent dissociation curve analysis relies on the use of well-designed primers that clearly amplify a single product. Furthermore, PCR products of different sizes may have similar melting temperatures. For example, *BM846 *amplified two products from river buffalo DNA, as shown in the agarose gel, but displayed a single dissociation peak (data not shown). We excised and sequenced both products visualized in the gel and calculated that the melting temperatures of the two products were within 1°C of each other, but amplicons did not appear to represent alleles of the same gene. Therefore, it may still be beneficial to test primer sets by visualizing control reactions via gel electrophoresis to ensure the presence of a single PCR product before proceeding with mapping.

## Conclusion

In summary, we developed a new method of marker scoring for RH maps. SYBR Green I-based qPCR and dissociation curve analysis offered greater sensitivity than the conventional gel-based scoring method, for detection of differences between buffalo and hamster products as well as reduction of scoring errors. To prove that this method can lead to better maps faster, we generated maps of river buffalo chromosome 20 using both old and new methods. The qPCR map contained 35% more markers and was based on a higher LOD score cutoff than the gel-based map. We have demonstrated that this novel application of dissociation curve analysis provides a reliable and efficient improvement over conventional methods for marker scoring in any species where RH mapping panels are available. Marker scoring remains the most labor-intensive step in RH map development. This sensitive method of qPCR-based marker scoring should enable faster construction of RH maps. As high-throughput methods are being developed for cross-species analysis, this method will remain a useful adjunct mapping tool.

## Methods

### Marker Selection

Fifty microsatellite markers and 9 genes or sequence tagged sites (STS) were selected to span the entire BTA21 chromosome map at intervals less than 5 cM. Most markers had published PCR annealing temperatures within 58–62°C, and were chosen from maps maintained by MARC , ENSEMBL , and NCBI . Six additional primer sets were designed with Oligo 6 software (Molecular Biology Insights, Inc., Cascade, CO) to anchor the RH map to the river buffalo cytogenetic map (*CHGA*, *SERPINA1*, and *GRP58*; [37]), and to increase the number of named genes on the map (*IGF1R, MESDC2 *and *MFGE8*). All markers were tested on DNA from a *B. taurus *bull (positive control for the markers), a river buffalo bull and Chinese hamster A23 cells (representing the donor and recipient species of the RH clones, respectively) by both qPCR and agarose gel analysis. Markers that amplified well in the river buffalo DNA and exhibited different dissociation curves between buffalo and hamster were used for typing the DNA from 90 RH panel clones [18].

### Real-time PCR and dissociation curve analysis

Real-time PCR was performed in a 20 μl reaction containing 20 ng template DNA, 1× *Power *SYBR Green PCR master mix (Applied Biosystems, Foster City, CA) and 300 nM primers. Amplification was carried out in 96-well plates in either a 7900 HT or a 7500 sequence detection system (Applied Biosystems) with the manufacturer's default thermal profile (50°C for 2 minutes, 95°C for 10 minutes, and 40 cycles of 95°C for 15 seconds and 60°C for 1 minute) followed by a dissociation stage (95°C for 15 seconds, 60°C for 15 seconds, followed by a slow ramp to 95°C). The incubation at 50°C was not necessary, but the instrument's default profile was intentionally not changed. For analysis in 384-well format, 10 μl reactions containing 10 ng template DNA were prepared with a Precision 2000 Plus automated microplate pipetting system (Bio-Tek Instruments, Inc., Winooski, VT) and amplified in a 7900 HT sequence detection system with the same thermal profile as described above. Amplification and dissociation data were analyzed with SDS software v.2.2.2 (Applied Biosystems) as described by Ririe et al. [29]. Radiation hybrid clones were scored independently by two people for presence or absence of the peak representing the river buffalo product. The scores were compared and discrepancies that were not clerical errors were scored as questionable.

### Agarose gel electrophoresis

Real-time PCR products were separated by electrophoresis on 2% SFR agarose gels (Amresco, Inc., Solon, OH). Digital images of the gels were captured using an Electrophoresis Documentation and Analysis System 290 (EDAS 290; Kodak, New Haven, CT). Gel images were scored in the same manner as, and independently from, the dissociation curve data.

### Sequencing

DNA was amplified by conventional PCR using 50 ng template DNA in a 50 μl reaction containing 3 U AmpliTaq Gold polymerase (Applied Biosystems, Foster City, CA), 3.0 mM magnesium chloride and 300 nM primers. Amplification was carried out in an ABI 2720 thermal cycler (Applied Biosystems) with an initial denaturation at 95°C for 5 minutes, followed by 35 cycles of 95°C for 15 seconds and 60°C for 1 minute. The products were purified through PSIΨClone PCR 96 columns (Princeton Separations, Adelphia, NJ). Sequencing was performed following PCR amplification in a 10 μl reaction containing 50 ng template DNA, 0.75× sequencing buffer, 500 nM primers and 1 ul BigDye v1.1 sequencing master mix (Applied Biosystems) with an initial denaturation at 98°C for 2 minutes, followed by 25 cycles of 96°C for 10 seconds, 50°C for 5 seconds and 60°C for 4 minutes. After purification through DyeEx spin columns (Qiagen, Valencia, CA) and resuspension in 10 μl deionized formamide, sequences of PCR products were determined with a 3130 × l Genetic Analyzer (Applied Biosystems) and data were analyzed with Sequence Analysis v5.2 software (Applied Biosystems). Melting temperatures of the PCR product sequences were calculated in Oligo 6 software.

### Map computation

For each of the two sets of marker scores, maps of BBU20 were computed using the maximum likelihood criterion with the software rh_tsp_map v3.0 ([24,31]; ) and CONCORDE [38] linked to Qsopt . The same general method was used to compute earlier maps of BBU chromosomes [18,22,28,30]. Frame markers passed a flips test at LOD threshold 0.5 and were assigned centiRay (cR) positions on the map. Some markers were placed in bins – intervals between frame markers – based on best order and passing flips at LOD threshold 0.5 within the bin, but with odds too low to establish cR positions. Markers that were not frame markers and could not be placed were dropped. In the qPCR map, markers *DIK3009 *and *BMS2382 *have identical scores and hence identical positions.

## Authors' contributions

KJK contributed to the design of the study, produced and analyzed RH scoring and sequencing data, and drafted the manuscript. MEJA provided the RH panel DNA, prepared data for map computation, and contributed to the design of the study. RA and AAS performed the mapping computations and helped draft the manuscript. PKR conceived and led the project, scored and analyzed RH data, and helped draft the manuscript. All authors read and approved the final manuscript.

## Supplementary Material

Additional file 1 **Markers tested but not used for mapping**. This table is a list of markers that were tested but not used for mapping. Click here for file

Additional file 2 **Comparison of marker typing and scoring by both methods**. A comparison of the number of markers found suitable for typing and scoring by either method is presented as a table in Additional File 2. Click here for file
