# Supplementary material for: Application of dissociation curve analysis to radiation hybrid panel marker scoring: generation of a map of river buffalo (B. bubalis) chromosome 20
Source: BMC Genomics. 2008 Nov 17;9:544. doi: 10.1186/1471-2164-9-544 (PMC2621213; doi:10.1186/1471-2164-9-544)
Supplement: Additional file 2 — Comparison of marker typing and scoring by both methods. A comparison of the number of markers found suitable for typing and scoring by either method is presented as a table in Additional File 2. [file 1471-2164-9-544-S2.doc]

Table S2. Comparison of marker typing and scoring by both methods.

|  | Real-time PCR/  Dissociation Curve | Conventional PCR/  Agarose Gel |
| --- | --- | --- |
| Markers tested | 65 | 65 |
| Markers that amplified well in buffalo | 40 | 37 |
| Markers not typed because primers amplified more than 1 fragment | 1 | 1 |
| Markers not typed because buffalo and hamster products were indistinguishable | 8 | 11 |
| Markers typed | 31 | 25 |
| Markers scored | 30* | 25 |
| Markers mapped | 27 | 20 |

**AKT2* was not scorable by dissociation curve analysis.
